# Controlled growth of ultrafine metal nanoparticles mediated by solid supports

**DOI:** 10.1039/d1na00025j

**Published:** 2021-02-15

**Authors:** Hongyin Hu, Shuanglong Lu, Ting Li, Yue Zhang, Chenxi Guo, Han Zhu, Yinghua Jin, Mingliang Du, Wei Zhang

**Affiliations:** Key Laboratory of Synthetic and Biological Colloids, Ministry of Education, School of Chemical and Material Engineering, Jiangnan University Wuxi 214122 Jiangsu China lushuanglong@jiangnan.edu.cn; Department of Chemistry, University of Colorado Boulder CO 80309 USA wei.zhang@colorado.edu

## Abstract

As a unique class of nanomaterials with a high surface-area-to-volume ratio and narrow size distribution, ultrafine metal nanoparticles (UMNPs) have shown exciting properties in many applications, particularly in the field of catalysis. Growing UMNPs *in situ* on solid supports enables precise control of the UMNP size, and the supports can effectively prevent the aggregation of UMNPs and maintain their high catalytic activity. In this review, we summarize the recent research progress in controlled growth of UMNPs using various solid supports and their applications in catalysis.

## Abstract 2

This article reviews the controlled growth of UMNPs mediated by different types of solid supports and their catalytic properties. The importance of certain structural features of the supports is also discussed.

## Graphical abstract

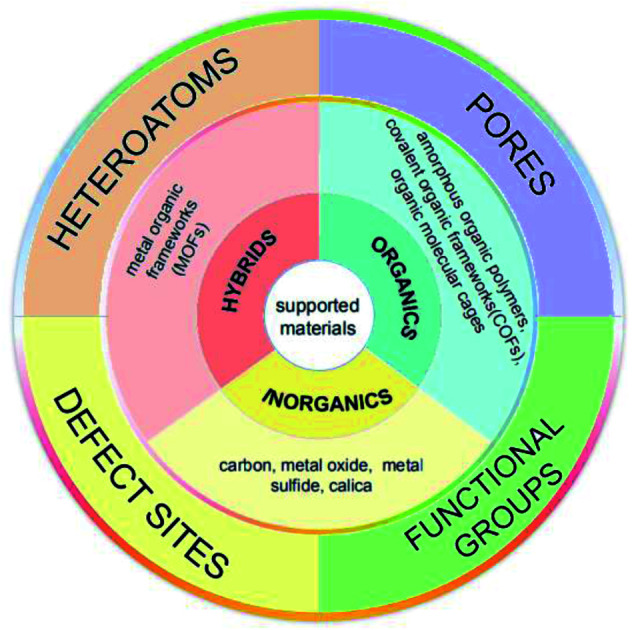

## 1. Introduction

Metal nanoparticles have fascinating properties applicable in many fields, such as catalysis,^1,2^ sensing,^3,4^ energy storage,^5,6^ and medicine.^7,8^ They have made significant contributions to the development of heterogeneous catalysts, showing excellent catalytic activity and reusability. During the catalysis, the adsorption and desorption of substrates occur on the surface of metal nanoparticles. The partially filled d-orbitals can promote the adsorption of reactants and catalyze the product formation at active sites. Therefore, the number of surface metal atoms plays a critical role in the catalytic process. The smaller the diameter of metal nanoparticles, the larger the corresponding specific surface area and the larger the number of surface atoms (active sites). When the diameter of metal nanoparticles is reduced from 10 nm to 1 nm, the “effective” accessible metal atoms will increase from 20% to 90%.^9^ Therefore, it is necessary to control the diameter of metal nanoparticles. Ultrafine metal nanoparticles (UMNPs), which usually refer to metal nanoparticles with an average size of less than 3 nm, have shown great application potential in the field of catalysis.^10^ Typically, UMNPs have a high surface area and narrow size distribution, which can provide a large number of available active sites for heterogeneous catalysis. Having a large number of surface atoms, UMNPs are thermodynamically unstable.^11–13^ Based on the migration-coalescence and Ostwald ripening mechanism, smaller particles aggregate to form larger particles through a spontaneous thermodynamic process.^14^ The aggregation leads to reduced catalytic activity of UMNPs. Maintaining the dispersion and stability of UMNPs throughout the catalysis is also an important issue that must be solved to realize widespread practical applications of UMNPs in catalysis.

Supports and organic capping agents are two commonly used methods for the preparation of UMNPs and preventing their aggregation.^2,10,15–17^ Organic capping agents, such as oleylamine and polyvinyl pyrrolidone, are often used to control the growth of metal nanoparticles.^16,17^ However, these capping agents usually adhere to the surface of UMNPs through strong interactions, blocking most of the catalytically active sites, thus significantly decreasing the catalytic activity of UMNPs. On the other hand, if the capping agent is removed, UMNPs tend to aggregate, which would affect their stability and also catalytic activity. Using solid supports to assist the UMNP synthesis can effectively solve the above problems. The supports can direct the growth of UMNPs and control their size through spatial confinement and electronic effects, so that different UMNPs can be prepared by varying the types and structures of supports.^16,18^ Moreover, by enhancing the interaction between MNPs and the support, the aggregation of MNPs can be minimized and the durability of the catalyst can be improved. Generally, metal ions or metal atoms whose d-orbitals are not filled can bind with electron-rich atoms on the support, such as S, N, O, *etc.*, through coordination bonds.^19–21^ Therefore, heteroatoms or organic functional groups provided by supports are usually required for the growth and stabilization of UMNPs. In addition to the electronic effect, the confinement effect of supports on UMNPs also makes a big difference. Particularly for porous materials with aligned or interconnected nanopores, the nanopores/channels can confine the growth of metal nanoparticles.^22,23^ Supports such as COFs, MOFs, and CMPs can be custom-designed and synthesized to achieve molecular-level control of the size of metal nanoparticles.^24–26^ Simply varying the chemical nature of solid supports, a series of UMNPs with high activity, high surface area and narrow size distribution can be obtained.

**Fig. 1:**
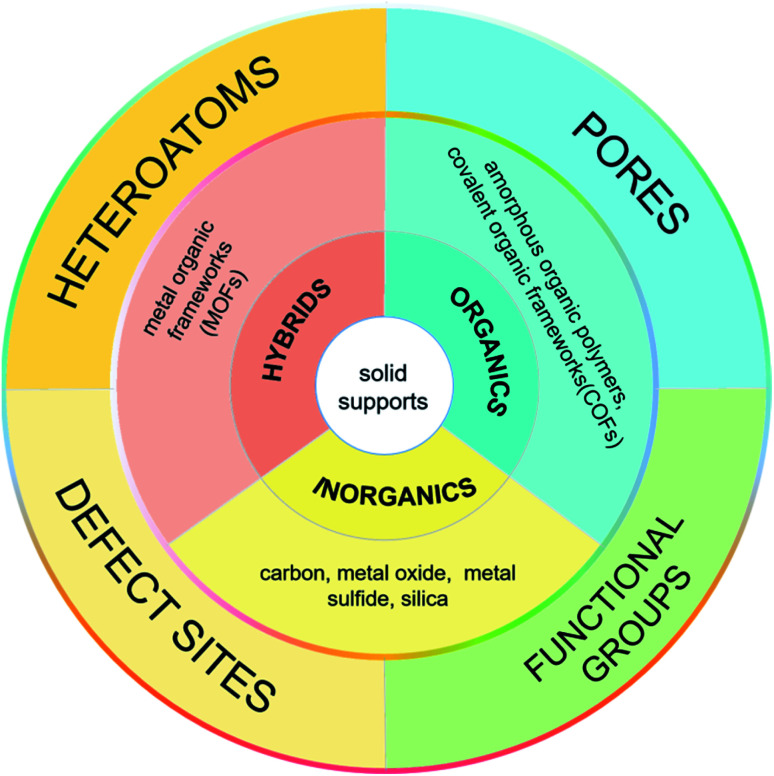
Classification of the solid supports used for controlled synthesis of UMNPs.

In 2016, Xu *et al.* summarized the preparation of UMNPs immobilized on solid supports and their applications in catalysis.^10^ They described the roles of different supports in detail and delivered a thoughtful review on the reported UMNPs mediated by solid supports. This review focuses on the solid supports (such as COFs, metal oxides/sulfides, *etc.*) and the recent new research progress that has not been not covered previously. We discuss the controlled nucleation and growth of UMNPs using different solid supports, aiming to reveal the influence of different supports (scaffolds) on the growth and performance of UMNPs. These studies have demonstrated that the solid supports can not only control the size of UMNPs and prevent their aggregation, but also improve their recovery and catalytic activity through electronic and spatial confinement effects (Fig. 1).

## 2. The role of solid supports and design principles for UMNP preparation

UMNPs have a large number of available active sites and excellent catalytic properties, but they tend to aggregate after cycles of catalytic reaction, resulting in a significant decrease in the number of available active sites. Using a suitable solid support to anchor and isolate the UMNPs from one another is a feasible approach for maintaining the dispersion of UMNPs. The work of synthesizing metal nanoparticles using closed-shell structures as scaffolds and loading Pd nanoparticles in organic porous polymers (POP) has been systematically summarized.^9,27^ The structural characteristics of the solid supports play an important role in the directed synthesis (*e.g.*, size and morphology) and stabilization of the resulting UMNPs. Spatial confinement and electronic confinement have dominated the variations of structural characteristics in solid supports.

### 2.1. The electronic confinement effects originated from the interaction between supports and UMNPs (or the metal ion precursor)

The nucleation and growth of UMNPs on the support are highly dependent on the nucleation site. The metal ion precursors will first nucleate at the anchoring site, triggering the nanoparticle growth.^28,29^ If the anchoring sites in solid supports are well distributed and provide strong interactions with the “seeds”, the resulting UMNPs would remain highly dispersed during the synthesis and maintain uniformity throughout the catalysis process. There are three common ways to introduce nucleation and anchoring sites into supports: installing organic functional groups, heteroatom doping and creating defect sites.^30–35^ Organic functional groups can bind the metal ions in the precursor solution. It is a common method to capture metal ions and anchor the resulting UMNPs in organic or hybrid supports, such as metal organic frameworks (MOFs) and covalent organic frameworks (COFs).^28,36^ For example, graphene oxide is a carbon support with abundant oxygen-containing groups, which can act as nucleation and anchor sites for UMNPs.^37^ Heteroatom doping changes the electronic properties of the supports and provides lone-pair electrons to form coordination bonds with the surface metal atoms of UMNPs. Suitable heteroatoms for anchoring UMNPs that have been reported include N, O, S, P and B atoms.^38–41^ These heteroatoms may not only provide nucleation sites for UMNPs, but also improve the electron transport between UMNPs and the supports. Different heteroatoms are usually chosen for different UMNPs. Defect sites are also used as traps to capture UMNPs and prevent their aggregation. The defect sites in carbon materials, metal oxide/sulfide and other materials can be designed to control the growth of and anchor the UMNPs.^42,43^ Embedding UMNPs in the solid supports could also enhance their surface area (thus accessible active sites) and improve their catalytic performance.

### 2.2 The spatial confinement effects originating from porous supports

The diameter of UMNPs loaded on the porous supports is highly dependent on the size of the pores, which enables the confined growth of UMNPs and prevents their overgrowth.^25,44^ By varying the diameter of the pores of the supports, the size of the UMNPs can be tuned. Such a confinement effect of the porous support can also prevent the aggregation of UMNPs.

## 3. Synthesis of UMNPs supported by carbon materials

Carbon materials are the most economical and most widely used supports in both industrial applications and fundamental research. Typical carbon materials include graphene, diamond, porous carbon and carbon nanotubes. All of them exhibit good chemical stability, high conductivity and low density, which are all favorable features when used as supports for UMNPs. However, carbon materials often lack sites for directly anchoring MNPs. Therefore, the use of carbon materials to support UMNPs often requires surface modification or heteroatom (such as N, S, P, O, *etc.*) doping to introduce the functionality onto the carbon support, which provides anchoring sites for UMNPs.^38–41^ At the same time, the doping of heteroatoms into the carbon materials can also effectively tune the properties of UMNPs anchored on the supports, further facilitating the catalytic reaction process and improving the catalytic efficiency.

### 3.1. Porous carbon

**Fig. 2:**
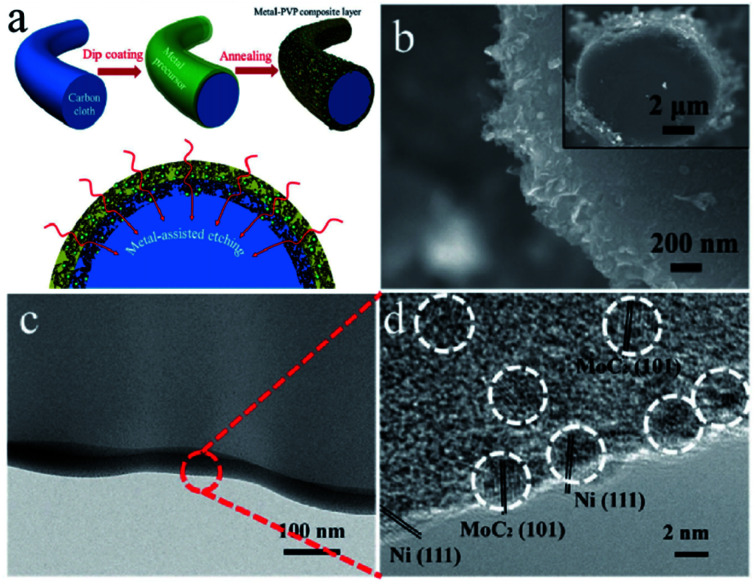
(a) Schematics of the formation of the metal-based nanoparticles/N-doped porous carbon hybrid catalysts; (b–d) characterization of NiMo UMNPs-N-doped porous carbon hybrid films: SEM (b) and TEM (c and d). Adapted with permission from ref. 45 @ copyright 2017 WILEY-VCH Verlag GmbH & Co. KGaA, Weinheim.

Porous carbon is an economical carbon material that has been mass-produced and widely used to support UMNPs. Porous carbon materials can be divided into microporous, mesoporous, and macroporous materials according to their pore size. According to International Union of Pure and Applied Chemistry (IUPAC) regulations, pores with diameters less than 2 nm are defined as micropores, those with diameters between 2 and 50 nm as mesopores, and those with diameters more than 50 nm as macropores.^18^ The inherent pores of porous carbon can be used as the accommodation sites of UMNPs generated *in situ* and prevent their agglomeration, which is beneficial to controlling the size of UMNPs. Microporous carbon usually has a large specific surface area, which can provide more anchoring sites for UMNPs. As shown in Fig. 2, Fan *et al.* coated microporous carbon fibers with metal precursors and polyvinylpyrrolidone (PVP).^45^ Metal-based nanoparticles/N-doped porous carbon hybrid films were obtained upon pyrolysis. In this process, PVP was converted into a N-doped carbon thin film, which provides binding interactions and confinement effects for UMNP formation. Nearly uniform UMNPs (2 nm) were observed. Carbon fibers were also etched by the metal at the high temperature to form micropores, increasing the specific surface area of the support with more exposed active centers. In addition, the nanoparticles are embedded in the carbon fibers. With the additional protection of microporous carbon, the UMNPs showed good stability in electrocatalysis, even at high current densities. However, the narrow channels of microporous carbon materials and the strong interactions between the solid support and the product or reactant resulted in slow mass transfer. By contrast, macroporous carbon materials have spacious pore channels, which facilitate the transportation of the product or reactant. However, the specific surface area of macroporous carbon materials is usually low with relatively limited anchoring sites. Besides, the pores of macroporous carbon are too large to provide suitable confinement for UMNPs. Therefore, porous carbon with only macropores is rarely reported as an efficient support for UMNPs. Mesoporous carbons have a relatively high specific surface area and spacious pore channels. Besides providing a large number of anchoring sites, they also possess relatively suitable pore channels for the mass transfer. Mesoporous carbons with ordered structures can be obtained by indirect template synthesis or direct synthesis. Yuan *et al.* have summarized the synthesis and modification methods of mesoporous carbons in detail.^46^ Nitrogen-doped mesoporous carbons with abundant anchoring sites for UMNPs represent one of the most promising substrates for catalysis. Wu *et al.* used nitrogen-doped mesoporous carbons as substrate materials and obtained Fe and FeC UMNPs through low-temperature liquid phase precipitation and heat treatment, which show excellent performance towards the electrocatalytic oxygen reduction reaction (ORR).^12^

**Fig. 3:**
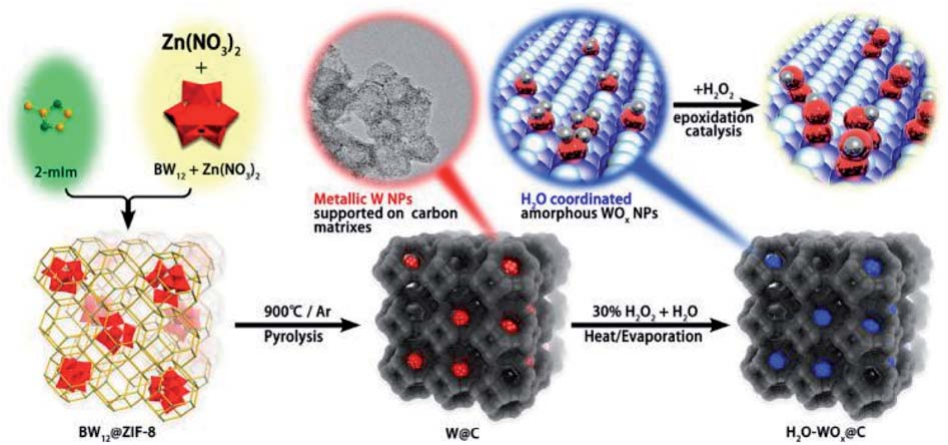
The structure of the MOFs after pyrolysis can protect UMNPs from aggregation (adapted with permission from ref. 48 @ copyright 2019 American Chemical Society).

In recent years, the carbonization of metal organic frameworks (MOFs) followed by chemical etching has emerged as one of the widely used approaches for preparation of mesoporous carbons. Zhang *et al.* prepared a mesoporous carbon support from the commercially available Cu-MOFs (HKUST-1) through the post-synthetic method. Such a solid support was used to prepared Pt UMNPs (2–3 nm), which have shown superior performance in both catalytic methanol oxidation and nitrophenol reduction reactions.^47^ Beyond the two-step post-synthesis, MOFs can also serve as hosts for various precursors of UMNPs, following which one-step co-pyrolysis can be used to fabricate the UMNPs@mesoporous carbon hybrid material. Niu *et al.* prepared tungsten UMNPs supported on a MOF-derived mesoporous carbon through carbonizing ZIF-8 consisting of encapsulated K_5_BW_12_O_40_.^48^ The catalysts show excellent performance towards olefin epoxidation. It is worthy of noting that Zn volatilizes at high temperature, leaving the W species supported by porous carbon. Although the pyrolysis process will destroy the fine structure of MOFs, the mesoporous carbon support after pyrolysis can still protect UMNPs from aggregation (Fig. 3).

To further optimize the electron and mass transfer during the catalytic process of UMNPs, hierarchical porous carbons, which contain two or three types of micropores, mesopores and macropores, are typically used.^49,50^ Hierarchical porous carbons combine a high specific surface area and wide pore channels to provide abundant anchoring sites for UMNPs. At the same time, rapid mass transfer can still be achieved. Therefore, hierarchical porous carbons are very promising supports for loading UMNPs for applications in heterogeneous catalysis. Zhong *et al.* introduced ethylenediaminetetraacetic acid (EDTA) into MOF-808.^28^ The EDTA acts as ion traps to capture Pt^2+^. The uncaptured Pt^2+^ ions form PtO_2_ clusters. Pt UMNPs loaded in porous carbon were obtained upon pyrolysis of the MOF precursors. With the increase of the Pt^2+^ concentration from 10 ppm to 80 ppm, the diameter of the obtained Pt particles was increased from 2.1 nm to 4.1 nm. When the concentration was reduced to 2 ppm, the particle diameter did not decrease, but the distribution density of Pt particles was reduced. This series of Pt UMNPs showed low overpotential in catalyzing the hydrogen evolution reaction (HER).

### 3.2. Hollow carbon

Hollow carbon structures, such as carbon tubes and hollow carbon spheres, are commonly used as carbon materials for UMNPs. Their unique hollow structures can provide superior spatial arrangement of anchoring sites, thus maximizing the utilization of UMNPs. Carbon nanotubes (CNTs) are typical hollow carbon structures, which have a high degree of graphitization, orderly arrangement of carbon atoms, excellent electrical conductivity and superior chemical stability.^51^ These characteristics make them one of the most ideal support materials in various applications. The standing disadvantage of CNTs lies in their lack of anchoring sites for UMNPs. Therefore, CNTs often need to be functionalized or doped with heteroatoms to improve their interactions with UMNPs.^52^

**Fig. 4:**
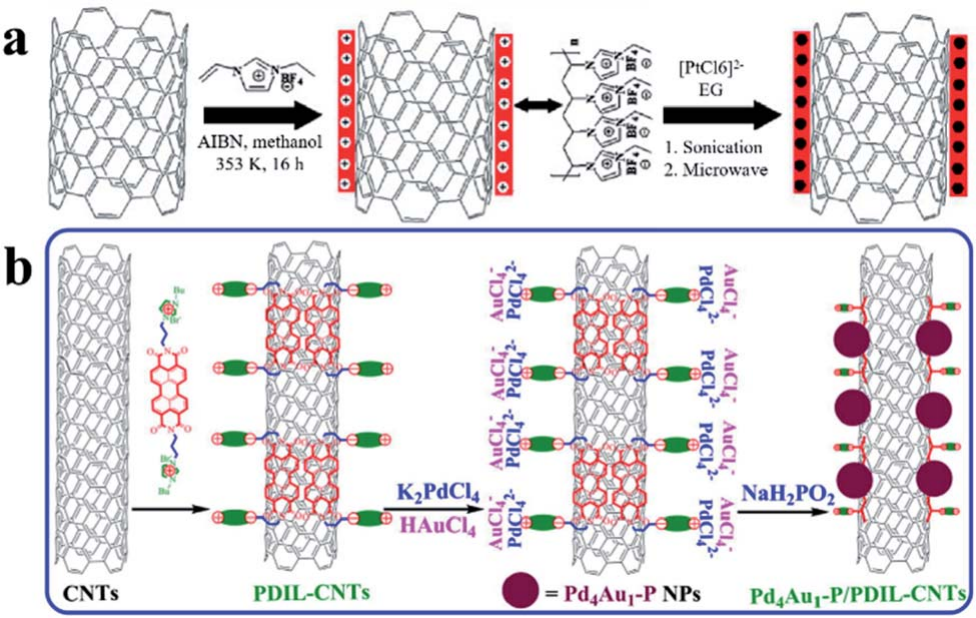
Functionalization of carbon nanotubes with an ionic liquid: (a) based on the thermal-initiation free radical polymerization of the ionic-liquid monomer 3-ethyl-1-vinylimidazolium tetrafluoroborate to form an ionic-liquid polymer on the CNT surface, which introduces a large number of surface functional groups onto the CNTs with uniform distribution to anchor and grow metal nanoparticles; (b) using ionic liquids, functional groups are non-covalently bound to CNTs with minimal change of the conductivity of CNTs (AIBN: 2,2′-azobisisobutyronitrile, EG: ethylene glycol, PDIL: 3,4,9,10-perylene tetracarboxylic acid) (adapted with permission from ref. 52 and 57 @ copyright 2017 Elsevier Inc. All rights reserved and 2009 Wiley-VCH Verlag GmbH & Co. KGaA, Weinheim).

There are many strategies for surface functionalization of CNTs, including oxidizing defect sites of CNTs, coating CNTs with polymers, and installing organic functional groups on the surface of CNTs covalently or non-covalently.^52–56^ The surface functionalized CNTs have more anchoring sites for better stabilization of UMNPs. Simply linking functional groups to CNTs through chemical oxidation or covalent bonds is the most feasible method to assist the growth of UMNPs on the surface of CNTs. Liu *et al.* used KMnO_4_, concentrated sulfuric acid, and H_2_O_2_ to oxidize CNTs in multiple steps, forming many defects and oxygen-containing groups on the surface of CNTs.^56^ These groups increase the dispersion of CNTs in aqueous solution and assist the formation of Ag UMNPs (∼3 nm) on the surface of CNTs. However, the oxidation process causes damage to the structure of CNTs and a significant decrease in electrical conductivity, limiting their application in electrocatalysis. Chen *et al.* used ionic liquid polymers to uniformly introduce abundant functional groups onto the surface of CNTs through radical polymerization (Fig. 4a).^52^ With the functionalized CNTs, uniformly distributed Pt (1.9 ± 0.5 nm) and PtRu (1.3 ± 0.4 nm) particles were successfully prepared on CNTs. The diameter of the particles loaded on non-functionalized CNTs was significantly larger, and the size distribution was wider for both PtRu (3.5 ± 1.0 nm) and Pt (5.5 ± 1.5 nm) nanoparticles. Similarly, as shown in Fig. 4b, Ma *et al.* used 3,4,9,10-perylene tetracarboxylic acid-derived ionic liquids to non-covalently functionalize CNTs. Pd_4_Au_1_–P nanoparticles (2.3 nm) supported on CNTs were obtained, which showed excellent ethanol oxidation (EOR) activity.^57^ Since the functional groups were non-covalently connected to CNTs through supramolecular forces such as π–π stacking, the electronic structure of CNTs remained intact and the excellent electrical conductivity of CNTs was reserved.

Incorporating heteroatoms into the framework of CNTs is also a common strategy for introducing UMNPs anchoring sites into CNTs, particularly the introduction of N atoms. Wang *et al.* reported a template method to fabricate home-made carbon tubes with uniformly doped N atoms.^58^ Different from conventional CNTs, these novel hollow carbon tubes are functionalized with a large number of binding sites for UMNPs. Highly dispersed Pd UMNPs (average size of 2.3 nm) were deposited on this N-doped carbon tube. Due to the synergistic effect of the support, Pd UMNPs can efficiently catalyze the reduction of 4-nitrophenol even at a low loading (Pd: 0.324 wt%).

**Fig. 5:**
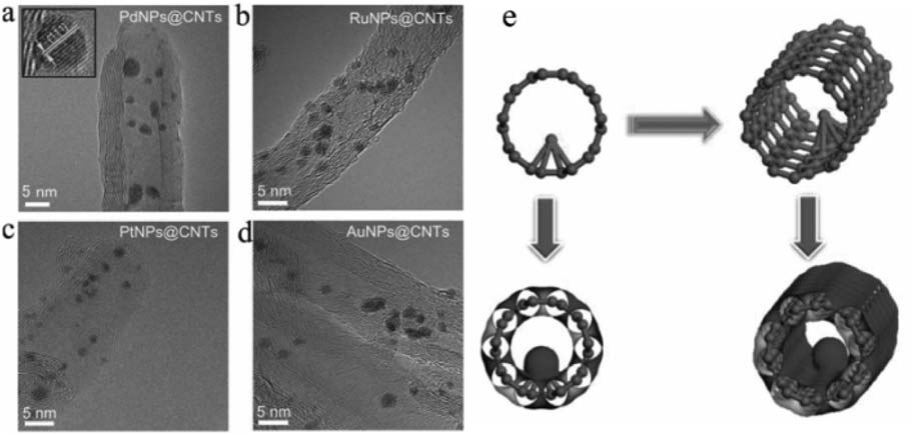
(a–d) HRTEM images of PdNPs@CNTs (a), RuNPs@CNTs (b), PtNPs@CNTs (c) and AuNPs@CNT (d); (e) schematic illustration of the formation of UMNPs@CNTs (adapted with permission from ref. 59 @ copyright 2014 WILEY-VCH Verlag GmbH & Co. KGaA, Weinheim).

Although carbon tubes with hollow structures have been widely used as catalyst supports, most of the UMNPs supported by carbon tubes are usually anchored on the outer surface of carbon tubes instead of the internal surface. Huang *et al.* encapsulated a series of noble metal UMNPs (Pd, Pt, Ru and Au) inside open-end CNTs by wet impregnation, followed by pyrolysis (Fig. 5).^59^ During the impregnation, metal ions enter CNTs through capillary action. When Pd UMNPs (2.5 ± 0.5 nm) were encapsulated into the CNTs, the resulting composite material showed excellent OER activity and can be used as a cathode material for Li–oxygen batteries. Other noble metal UMNPs, including Ru UMNPs (2.7 ± 0.5 nm), Pt UMNPs (1.8 ± 0.5 nm) and Au UMNPs (3.0 ± 0.5 nm) were also successfully encapsulated into CNTs.

**Fig. 6:**
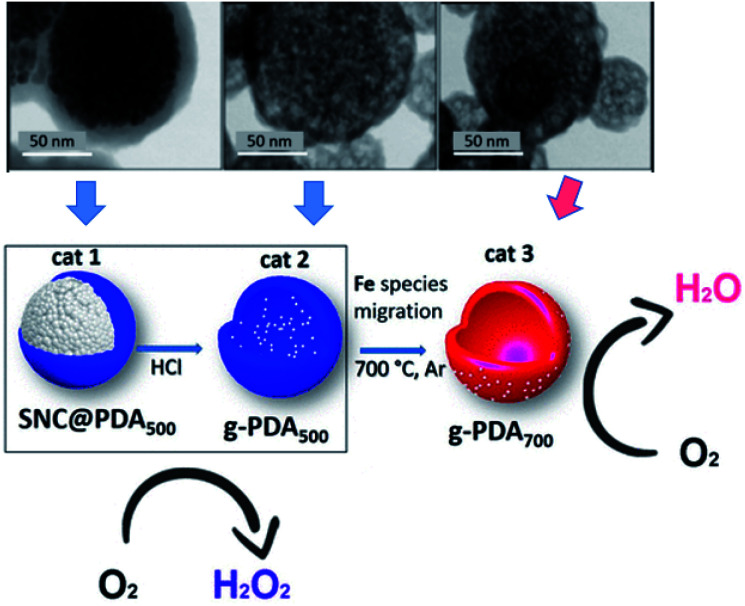
Under different pyrolysis conditions, FeO_*x*_ particles were formed on different locations of CNTs as shown in the TEM images. Correspondingly, different ORR product selectivities were observed (SNC: superparamagnetic nanoparticle clusters, PDA: polydopamine) (adapted with permission from ref. 61 @ copyright 2019 American Chemical Society).

Similar to carbon tubes, hollow carbon spheres (HCNs) with a unique hierarchical structure of a spherical shell represent another type of hollow carbon material, which also has highly exposed anchoring sites for UMNPs. Nanda *et al.* reported PtPd–UMNPs (2 nm) dispersed in heteroatom-doped HCNs, which showed excellent catalytic performance and stability in the methanol oxidation reaction (MOR), EOR and ORR.^60^ The high surface area of UMNPs brings about high catalytic activity, and the surrounding carbon layer prevents the migration and aggregation of UMNPs. The carbon structure also adsorbs a small amount of –OH/–H_2_O species, which is conducive to the removal of CO produced during the reaction, particularly in the MOR, to reduce the risk of catalyst poisoning. Fornasiero *et al.* reported a new synthetic approach to prepare FeO_*x*_ UMNPs on the surface of HCNs through an “inside-out” mechanism.^61^ They controlled the pyrolysis conditions, which can exsolve Fe species from the inside of the HCNs to the outside and form FeO_*x*_ UMNPs. The different location (inside or outside) of FeO_*x*_ led to the different selectivity (form H_2_O or H_2_O_2_) in the ORR (Fig. 6).

### 3.3. Graphene and its derivatives

Graphene is a two-dimensional carbon nanomaterial with a hexagonal honeycomb lattice composed of carbon atoms and sp^2^ hybrid orbitals. In theory, it has a huge specific surface area up to 2600 m^2^ g^−1^. Graphene has excellent optical, electrical, and mechanical properties and has important application prospects in materials science, micro–nano processing, energy, biomedicine, and drug delivery.^62,63^ Graphene oxide (GO) is an important derivative obtained through oxidation of graphene. It has a large number of oxygen-containing functional groups, including hydroxyl, epoxy, carbonyl, carboxyl, *etc.*, which are favorable anchoring sites for metal ions and UMNPs. Reduced graphene oxide (rGO) is obtained by reduction of GO. Compared with graphene, rGO not only has residual oxygen and other heteroatoms, but also has some defects, which can act as barriers for atom migration and maintain the uniform distribution of UMNPs.^64^

**Fig. 7:**
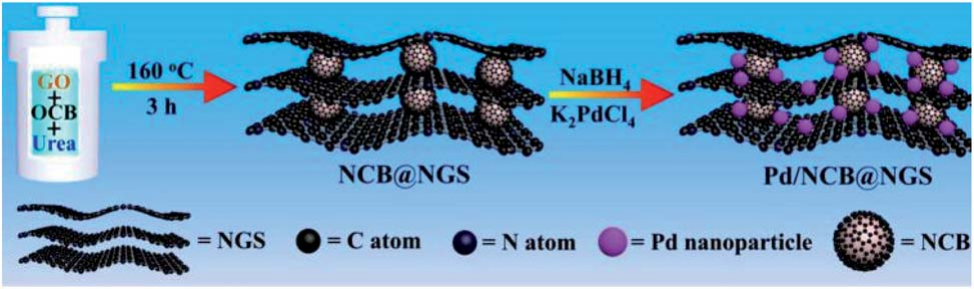
Using spherical carbon black as the gap between GO layers to maintain GO dispersion. (NCB: nitrogen-doped carbon black, NGS: nitrogen-doped graphene) (adapted with permission from ref. 68 @ copyright 2020 Elsevier B.V. All rights reserved).

Graphene only shows good dispersibility in non-polar solvents, which limits its use due to the fact that most metal precursors are dissolved in polar solvents.^64^ In addition, the uniform structure of graphene lacks the anchoring sites for UMNPs. In contrast to graphene, GO can be easily dispersed in water or polar organic solvents. The oxygen-containing groups of GO can facilitate exfoliation of graphene nanosheets and prevent graphene stacking. GO can be easily produced at low cost, but strong oxidants are inevitably used in the GO production process, resulting in a large number of defects in the GO network. It causes the conductivity of GO to be far inferior to that of graphene. rGO exhibits conductive properties similar to graphene and contains a small amount of unreduced oxygen-containing functional groups and defect sites.^65^ The most convenient way to anchor UMNPs on GO is to reduce the metal precursor and GO at the same time. Then, the metal precursor will be reduced to form UMNPs while GO transforms into rGO. Ravishankar *et al.* used ethylene glycol to simultaneously reduce Pt^2+^ and GO under microwave irradiation conditions and load Pt UMNPs (2–3 nm) on graphene.^66^ Liang *et al.* reported the formation of uniformly dispersed Cu UMNPs (2 nm) on rGO through laser ablation of a Cu target in GO solution followed by reduction treatment.^67^ The obtained Cu UMNPs showed excellent performance towards 4-nitrophenol reduction. However, the reduction process of metal precursors also reduces the oxygen-containing functional groups on GO. To prevent GO stacking due to the loss of oxygen-containing functional groups, Jin *et al.* introduced spherical carbon black as the gap between GO layers to form a stable 3D structure and maintain the high specific surface area of GO (Fig. 7).^68^ The carbon black and graphene doped with nitrogen were obtained upon reduction with urea. They used such a unique three-dimensional structure as the support for the growth of Pd UMNPs. Both the incorporated N sites and the initial oxygen-containing functional groups serve as the binding sites for Pd ions. Upon NaBH_4_ reduction, Pd UNMPs (3.2 nm) grew *in situ* in the retained 3-dimensional composite structure of rGO and carbon black. The hybrid catalysts showed excellent activity towards the electrocatalytic ethanol oxidation reaction.

## 4. Synthesis of UMNPs supported by crystalline frameworks

Metal organic frameworks (MOFs) and covalent organic frameworks (COFs) are emerging porous materials with rigid frameworks and orderly lattice fringes. Unlike conventional crystalline materials, these framework materials are constructed from organic building blocks or through hybridization of organic building blocks and metal ions. By selecting different organic monomers, porous crystalline frameworks with different pore sizes, morphologies and functional groups can be synthesized. These materials typically have great structural tunability and high porosity, making them ideal catalyst support materials.^69–71^ Apart from being precursors for porous carbon which has been discussed in Section 3.1, the rigid frameworks provide stable pores without collapse for a long time, and the backbones between the cavities can prevent the agglomeration of UMNPs.

### 4.1. MOFs

MOFs are a class of organic–inorganic hybrid materials with intramolecular pores formed by self-assembly of organic ligands and metal ions or clusters through coordination bonds. MOFs have highly tunable pore size and volume and an easily modifiable inner surface.^69^ In addition, their frame structure is rigid and not easy to collapse. Moreover, MOFs usually have good thermal stability. These exciting features make MOFs very promising support materials for UMNPs. Through rational design, metal ions or UMNPs can be precisely and uniformly coordinated and deposited on MOFs.^72^ The pore structures of MOFs separate the UMNPs one from another, which can avoid the aggregation of UMNPs during the catalytic process.^73,74^ The synthesis and application of MOFs is a current hot research field, and many new MOF structures are reported every year, but not all MOFs are suitable for the synthesis of UMNPs and their application in catalysis. For example, when H_2_O is generated or participates in the catalytic reaction pathway, the framework of MOF-177 can easily decompose and collapse.^75^ MOFs with suitable pore structures and high stability are required for the confined growth of UMNPs for catalysis application. Xu *et al.* have reviewed the controlled synthesis of UMNPs by using MOFs as supports in detail.^10^ Therefore, we will only briefly discuss the typical cases of the controlled synthesis of UMNPs with MOFs in recent years in this section.

**Fig. 8:**
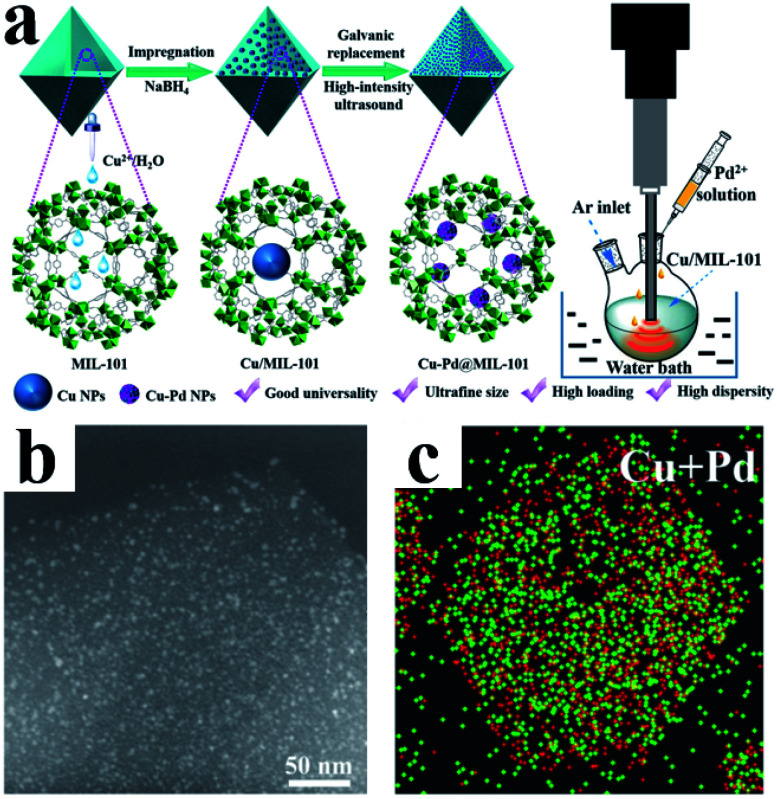
(a) Preparation of Cu–Pd@MIL-101; (b) HAADF-STEM image and (c) elemental mapping of PdCu UMNPs@MIL-101 (adapted with permission from ref. 24 @ copyright 2019 American Chemical Society).

Li *et al.* demonstrated a method of using MIL-101 as a template to fix a variety of bimetal alloyed UMNPs in the pores (Fig. 8).^24^ The particle diameter was 1.1–2.2 nm, and the metal loading was up to 10.4 wt%. Taking Cu–Pd@MIL-101 as an example, they first introduced Cu^2+^ into MIL-101 by dipping and then reduced them with NaBH_4_ to obtain Cu particles with a diameter of 8.25 ± 2.53 nm. Subsequently, the Pd precursor was introduced, and under the action of high-intensity ultrasound, the Cu particles were effectively transformed into Pd–Cu UMNPs (diameter of about 2.16 nm) through the electric substitution reaction with Pd^2+^. The catalyst was used to catalyze the coupling of phenylacetylene to produce 1,4-diphenylbuta-1,3-diyne in excellent yield (up to 98%).

The heteroatoms incorporated into the MOF backbone or side chains can act as nucleation and anchor sites just like carbon materials. The surface micro-environment of UMNPs can be modulated by changing the functional groups and metal substitution in the MOFs to enhance their catalytic performance. Jiang *et al.* encapsulated Pd UMNPs (less than 1.1 nm) into the pores of UiO-66-X (X = H, OMe, NH_2_, 2OH, 2OH(Hf/Zr)): X represents the tunable organic groups linked to the benzene ring, Hf/Zr represents the Hf-oxo or the Zr-oxo cluster.^76^ Different organic linked groups in the pores of the MOFs can change the adsorption energy of Pd@UiO-66-X to a substrate and the charge transfer between Pd UMNPs and MOFs. The catalytic activity of Pd@UiO-66-2OH was around 14 times higher than that of Pd@UiO-66 in the hydrogenation of benzoic acid. In addition, changing the metal clusters (2OH, 2OH(Hf)) also led to different catalytic performances. The authors highlighted the influence of the surface microenvironment on the guest metal NPs, which can be tuned by choosing different substituents of the metal and functional groups in the host MOFs.

**Fig. 9:**
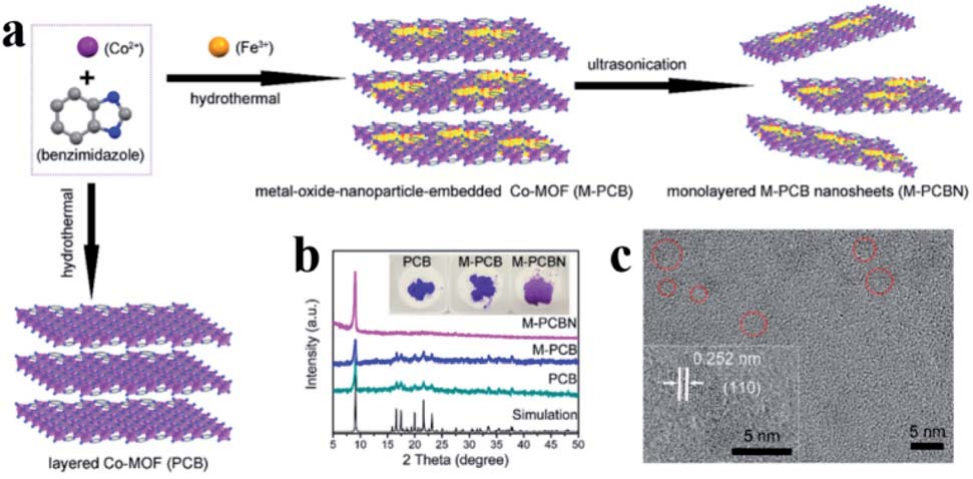
(a) Schematic of embedding FeO in the lattice of the MOF; (b) powder XRD patterns of PCB, FeCoO_*x*_ UMNPs-PCB, and FeCoO_*x*_ UMNPs-PCBN; and (c) HRTEM image of FeCoO_*x*_ UMNPs-PCBN (adapted with permission from ref. 77 @ copyright 2020 American Chemical Society).

Embedding UMNPs into the lattice of MOFs can enhance the dispersibility of UMNPs. The electronic configuration at the interface between UMNPs and MOFs can be tuned, which may increase their catalytic performance. Zhou *et al.* used cobalt and benzimidazole to synthesize a MOF, (poly-[Co_2_(benzimidazole)_4_] (PCB)), with a laminar flow structure and introduced Fe^3+^ into the pores of the MOF by dipping (Fig. 9).^77^ After reduction, the generated FeCoO_*x*_ UMNPs (3 nm) were embedded into the lattice of MOFs. When deposited on carbon cloth, such a composite material showed excellent activity in catalyzing the oxygen evolution reaction (OER).

### 4.2. COFs

COFs are a class of 2D or 3D crystalline organic polymers connected by covalent bonds. Similar to MOFs, COFs also have highly tunable pore size and easily modified inner surface. Therefore, the introduction of suitable functional groups into the constituent units of COFs can equip COFs with binding sites for UMNPs.^78,79^ Compared with MOFs, COFs have lower density without any metals in their frameworks. In addition, they generally exhibit higher stability in acid, alkali, organic and aqueous media when compared with MOFs.

**Fig. 10:**
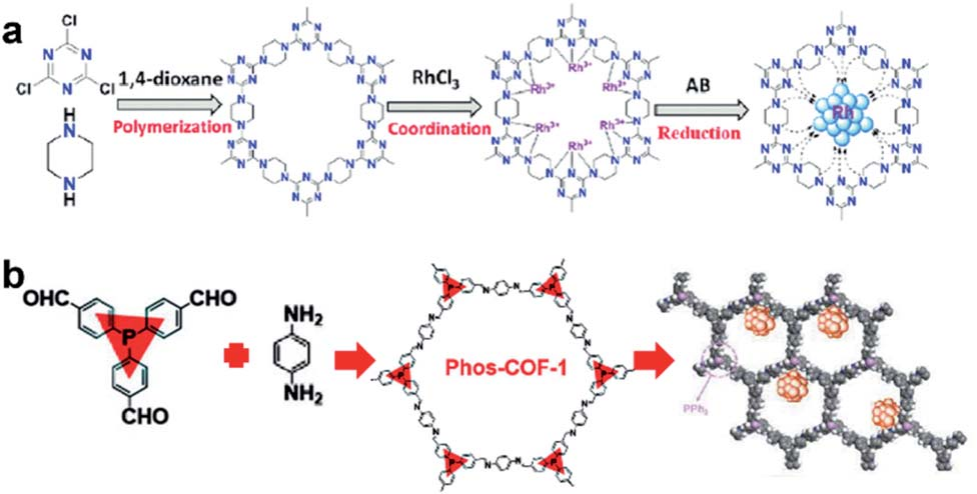
Synthesis of COF supported UMNPs: (a) N acts as the nucleation and growth sites for Ru UMNPs; (b) PPh_3_ provides anchoring sites for UMNPs@Phos-COF-1 (adapted with permission from ref. 33 and 80 @ copyright the Partner Organisations 2020 and 2020 WILEY-VCH Verlag GmbH & Co. KGaA, Weinheim).

The introduction of anchoring sites into COFs has been mainly accomplished in two ways: (1) incorporating the sites into the backbones of COFs and (2) installing the sites as side chains on the COF backbones. The N-rich COF backbones have a large number of N atoms, which can provide abundant anchoring sites for UMNPs. Through the coordination of N atoms and the confinement of intrinsic pores, UMNPs can be well encapsulated and the aggregation of UMNPs can be prevented. A series of COFs with N-rich backbones have been successfully developed using synthetic building blocks with nitrogen-containing functional groups such as carbazole, porphyrin, amide and imine. Lu *et al.* used piperazine and cyanuric chloride as molecular building blocks to synthesize a COF with the N-rich backbone that can coordinate Ru ions (Fig. 10a).^80^ Ru ions were reduced *in situ* to Ru UMNPs (1.4 to 2.6 nm), which were embedded in the pores of the COF. The confinement effect from the pores and the coordination with N atoms firmly anchored the UMNPs in the COF framework. The obtained UMNPs were used to catalyze the methanolysis of ammonia borane, delivering a total turnover frequency (TOF) up to 505 min^−1^ at 298 K. Moreover, the catalyst showed excellent durability and maintained high dispersion during the catalytic process.

In addition to nitrogen-containing functional groups, other heteroatom-containing functional groups can also be used as anchor sites to coordinate UMNPs. Phosphine groups have been widely used as organic ligands for metal organic catalysts. Incorporating phosphine groups into COFs can make the phosphine groups distributed in an orderly manner and provide anchor sites for metal ions or UMNPs. Recently, a COF material (Phos-COF-1) that incorporates triphenylphosphine (PPh_3_) as a growth site for UMNPs was reported.^33^ A series of UMNPs (Pt, Pd, Au and PdAu) with narrow size distribution were successfully prepared inside Phos-COF-1 (Fig. 10b). The loading of metal precursors is also an important factor affecting the size of UMNPs. Excessive metal loading promotes the excessive random growth of UMNPs on the surface of COFs. Taking the Pd UMNPs synthesized in this work as an example, after doubling the amount of the Pd precursor, the diameter of Pd UMNPs changed from 1.62 nm to 2.74 nm on average and the size distribution range became wider. The obtained catalysts were used to catalyze a number of reactions including nitrophenol reduction, cross coupling, and tandem cross coupling–nitrophenol reduction. All of these catalysts exhibit excellent performance.

Besides directly anchoring UMNPs through the sites in the backbone of COFs, COFs can also be functionalized in their side chains to improve the binding ability towards metal ions or particles. Post synthesis and bottom-up synthesis are the two commonly used strategies to install functional groups.^81–83^ The post synthesis avoids functional groups participating in the polymerization of building blocks to form COF backbones. However, through post-synthesis, the crystallinity of COFs may be reduced and not all pores can be fully functionalized. The advantage of bottom-up synthesis lies in the well-maintained crystallinity of COFs and the functional groups can be evenly distributed in the framework. However, some functional groups may either be unstable under severe synthetic conditions of COF formation or interfere with the formation of crystalline COF structures.

**Fig. 11:**
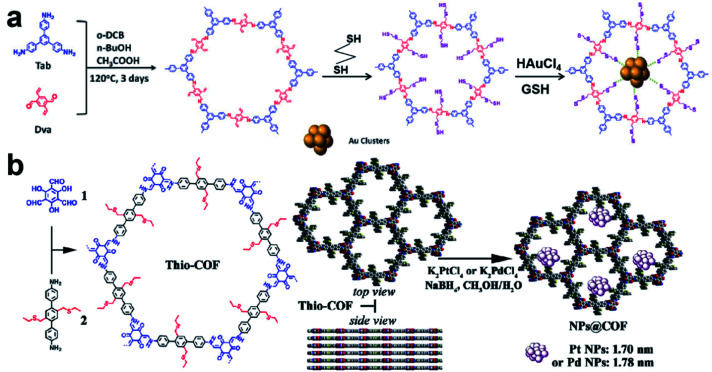
(a) Installation of thio groups through the post-synthetic approach: synthesis of Au@COF (*o*-DBC: 1,2-dichlorobenzene, GSH: glutathione); (b) installation of thio groups through the bottom-up synthetic approach: synthesis of Thio-COF and schematic representation of the synthesis of Thio-COF supported PtNPs@COF and PdNPs@COF (adapted with permission from ref. 25 and 84 @ copyright 2020 Wiley-VCH Verlag GmbH & Co. KGaA, Weinheim and 2017 American Chemical Society).

Recently, Lu *et al.* reported a COF containing thiol groups (–SH) *via* post-synthesis strategies (Fig. 11a).^84^ The small pores of COFs and the strong S–Au binding energy enabled the confined growth of Au UMNPs with narrow size distribution (1.8 ± 0.2 nm). Such a composite material showed enhanced photostability and photocatalytic performance in the photodegradation of RhB. The synthesis of a COF containing thioethers (Thio-COF) using bottom-up strategies was also reported (Fig. 11b).^25^ Pt UMNPs (1.70 nm) and Pd UMNPs (1.78 nm) confined in Thio-COF were obtained, which showed excellent catalytic activity towards the reduction of 4-nitrophenol and the Suzuki–Miyaura coupling reaction, respectively. The thioether group with strong binding capability for metal ions or nanoparticles was pre-installed in the building block. It has been proposed the thioether groups serve as nucleation centers for the confined growth of UMNPs in COFs. When COFs without thioether in their channels were used as supports, randomly agglomerated metal particles were formed instead. In addition, the use of an amorphous COF with the same structure as the support also led to random distribution of particles. Therefore, both the crystalline structure of COFs and the suitable functional groups in the channels contribute to the high quality of the as-formed UMNPs. Similar studies using bottom-up synthesis of functionalized COFs for UNMPs have also been reported for Pd UMNPs@COF, Fe–TiO_2_ UMNPs@COF.^19,85^

## 5. Synthesis of UMNPs supported by amorphous organic polymers

In addition to ordered COFs, there are some other disordered polymer materials, which can also be used as supports to direct UMNP growth. Their design concept is similar to COFs, that is, modifying the polymer backbone with organic functional groups or incorporating heteroatoms.^86^ They can also provide a confined environment for the growth of UMNPs. Several common amorphous polymers that can be used to control the synthesis of UMNPs include conjugated micro-/meso-/macro-porous polymers (CMPs), hyper-crosslinked polymer (HCPs) and other porous organic polymers (POPs).

**Fig. 12:**
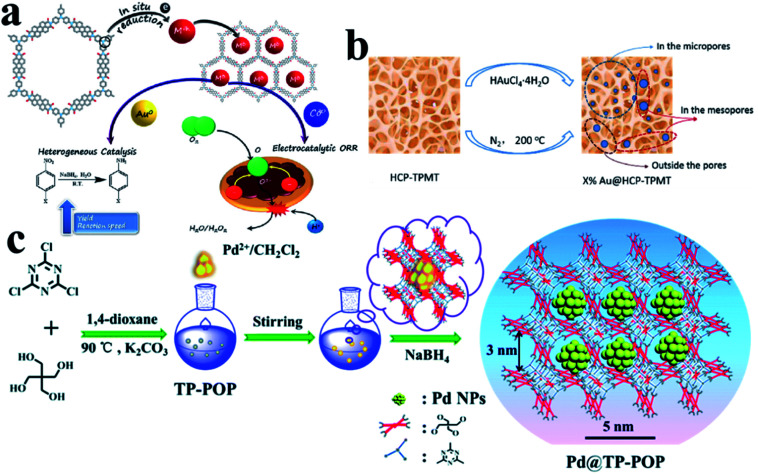
Controlling the growth of UMNPs using amorphous polymers: (a) Au@CMPs and Co@CMPs; (b) Au@HCPs; (c) Pd@TP-POP (adapted with permission from ref. 22, 32 and 89 @ copyright 2019 American Chemical Society, 2018 American Chemical Society and The Royal Society of Chemistry 2018).

CMPs have π-conjugated main chains and permanent porous structures, which distinguishes them from unstable porous materials, non-porous conjugated polymers, and porous carbon materials. By choosing different molecular building blocks, CMPs with different pore sizes and morphologies can be obtained.^87^ It is worth noting that because imine bonds can contribute to conjugation, certain imine-based COFs can also be considered as crystalline CMPs. CMPs can also be considered amorphous analogs of COFs.^88^ The conjugated structures of CMPs improve the electron transfer efficiency, which helps in electron transport in the catalysis process. It is even anticipated that it could be used directly in electrochemical catalysis. The work of Maji *et al.* gave an example of loading UMNPs onto CMPs and directly using them in the electrochemical catalysis (Fig. 12a).^89^ They used tris-(4-aminophenyl)amine (TPA) and perylenedianhydride (PDI) as raw materials to synthesize a CMP material with good conductivity, called TPA–PDI. Triphenylamine and its derivatives are well-recognized hole-transport agents. When coupled with electron acceptor building blocks, they can produce a CMP system with inherent conductivity. The special donor–acceptor pair and conjugation of TPA–PDI facilitate the convenient transfer of charges across the CMP structure, which makes this material promising for application in electrocatalysis. They used TPA–PDI to synthesize two UMNPs (Au and Co), called Au@TPA–PDI and Co@TPA–PDI, respectively. Au@TPA–PDI can efficiently catalyze the reduction of nitroaromatics to aminoaromatics. More interestingly, both TPA–PDI and Co@TPA–PDI exhibit considerable ORR catalytic activity without carbonization. Recently, Ag UMNPs@CMPs, Pd UMNPs@CMPs and others have also been reported.^26,90^

HCPs are a class of microporous polymers that are prepared by extensive crosslinking of linear or lightly crosslinked precursor polymers. They have high surface area, high porosity and high stability, which make them the candidate materials to support UMNPs.^91^ The heteroatom, organic functional groups and hyper crosslinked tubes in HCPs can prevent UMNPs aggregation.^92^ Dong *et al.* prepared thiol-containing HCPs using ferrocenecarboxaldehyde and melamine as building blocks, which were loaded with Pd UMNPs (2.89 nm).^93^ The resulting Pd UMNPs@HCP composites showed excellent activity and stability in the catalytic reduction of nitroarenes. Tan *et al.* reported a series of Au UMNPs (range of 1.7–5.1 nm) supported on a HCP (Fig. 12b).^22^ By simply varying the mass ratio of HAuCl_4_·4H_2_O and the HCP, the Au UMNPs with different sizes can be obtained. These composite materials exhibit high catalytic activity and recyclability toward catalytic reduction of 4-nitrophenol.

Wang *et al.* presented TB-POP containing triphenylamine and 2,6-bis(1,2,3-triazol-4-yl)pyridyl units.^31^ Adding TB-POP into CH_2_Cl_2_ solution to adsorb palladium acetate followed by reduction in a stream of H_2_/N_2_ at 200 °C provided a composite material with Pd UMNPs (1.5 ± 0.6 nm)/TB-POP. Pd UMNPs uniformly dispersed over TB-POP exhibit remarkable catalytic activity and high selectivity in the dehydrogenation of aqueous formic acid solution. Dong *et al.* fabricated highly dispersed Pd UMNPs (1.4–2.8 nm) loaded on a stable triazinyl-pentaerythritol porous organic polymer (TP-POP) which was synthesized through a facile polycondensation approach between the cyanuric chloride and pentaerythritol (Fig. 12c).^32^ The three-dimensional structure, abundant triazinyl groups and abundant pores control the growth of Pd UMNPs and prevent their agglomeration during the catalytic process. The as-prepared Pd UMNPs@TP-POP catalyst showed excellent catalytic activity and stability in the reduction of 4-nitrophenol and transfer hydrogenation of aromatic aldehydes under mild conditions.

## 6. Synthesis of UMNPs supported by metal oxide/sulfides

Metal oxides/sulfides (MX_*n*_, X = O or S) are also proved to be efficient support materials for UMNPs. Vacancies or defects are usually introduced into MX_*n*_ to promote the interactions between the support and metal atoms or particles.^94^ Such strong binding interactions help UMNPs anchor in MX_*n*_ tightly, stabilizing UMNPs and preventing their aggregation. In addition, oxygen vacancies in metal oxides have oxygen storage capacity, which can play an important role in promoting oxygen involved reactions, such as oxygen evolution, oxygen reduction and CO oxidation.^95–97^ Moreover, many MX_*n*_, such as CdS and TiO_2_, can not only serve as supports for UMNPs, but also have catalytic activity by themselves, synergistically acting as co-catalysts.

**Fig. 13:**
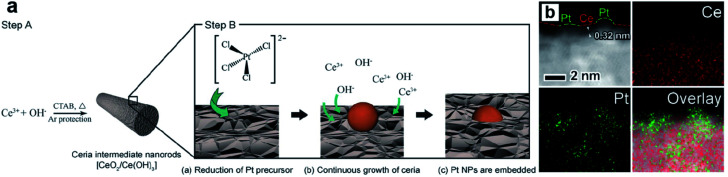
(a) Schematic illustration of the proposed mechanism of synthesizing surface-embedded Pt/CeO_2_ hybrid nanorods and (b) HAADF-STEMEDX mapping images of Pt/CeO_2_ hybrid nanorods (adapted with permission from ref. 98 @ copyright 2017 WILEY-VCH Verlag GmbH & Co. KGaA, Weinheim).

Zhang *et al.* developed a way of *in situ* embedding Pt UMNPs (1–2 nm) into a nanorod-shaped cerium dioxide (CeO_2_) through a redox reaction that occurs at the solid solution interface (Fig. 13).^98^ CeO_2_ has extremely high oxygen storage performance and strong interactions with metals; therefore, is often used as a support for MNPs. Pt–CeO_2_ shows superior thermal stability and durability because of the strong Pt–O bonds formed between Pt UMNPs and O atoms in CeO_2_. The enhanced interface between Pt and CeO_2_ is also responsible for the excellent catalytic performance of the hybrid catalyst towards the hydrogenation of nitrophenol. Jiang *et al.* used Pt ion-doped Ce-MOFs to create Pt UMNPs (<2 nm) supported on CeO_2_ particles (s-Pt/CeO_2_) through ultrafast laser induced reduction.^34^ In this process, the MOF crystals absorb laser photons and generate high pressure and high temperature, causing the pyrolysis of organic liners and reduction of metal ions. Nanocrystalline CeO_2_ particles with abundant defects on their surface were *in situ* precipitated, which serve as anchoring sites for Pt UMNPs. Oxygen vacancies in the CeO_2_ support often play a role in promoting CO oxidation. As expected, the s-Pt/CeO_2_ can quickly convert CO to CO_2_ with a conversion efficiency up to 100%.

The porous structures of supporting materials can increase the reaction rate by accelerating the mass transfer process of the catalytic reactions and improve the stability of UMNPs as well. Various metal oxides are featured as porous structures, which serve as ideal supports for UMNPs.^99–101^ Zhan *et al.* supported Pt UMNPs (∼3.2 nm) on mesoporous TiO_2_ and used them to catalyze the ORR.^102^ The strong metal support interaction between Pt UMNPs and porous TiO_2_ enhanced the stability of the catalyst, together with the spatial restriction and the anti-restriction provided by mesoporous TiO_2_. The catalyst exhibited a much higher stability than the commercial Pt/C after 10 000 cycles. Meijboom *et al.* reported Pt UMNPs (1.1–2.1 nm) on mesoporous Co_3_O_4_ for the catalytic oxidation of methylene blue.^103^ The similar mesoporous Co_3_O_4_ was also applied by Dai *et al.* to support AuPd UMNPs (2.7–4.5 nm) for catalytic methane combustion.^104^ In addition to improving the stability of catalysts, mesoporous Co_3_O_4_ provided a high adsorbed reactant concentration due to its porous structure and high pore volume.

**Fig. 14:**
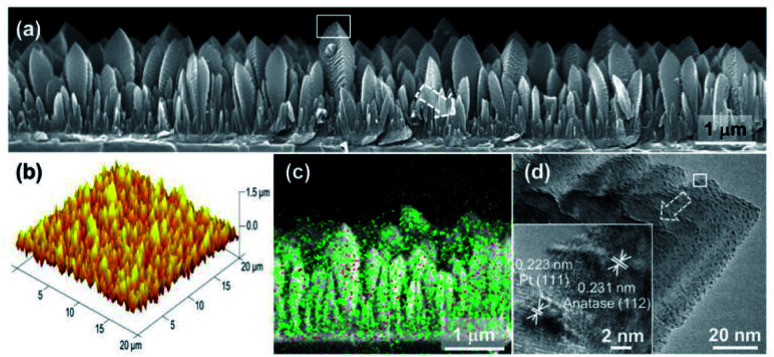
Characterization of a columnar TiO_2_ thin film loaded with Pt UMNPs (adapted with permission from ref. 42 @ copyright 2012 American Chemical Society).

Loading UMNPs on some photocatalytic catalysts, such as TiO_2_, is also a good approach for improving specific photocatalytic activity. In many cases, the low photocatalytic conversion efficiency is due to the rapid electron–hole recombination in the photocatalyst. Pt NPs are believed to delay electron–hole recombination by capturing electrons and promoting interface electron transfer. Biswas *et al.* obtained a TiO_2_ film through the aerosol chemical vapor deposition route and deposited Pt UMNPs (0.5–2 nm) on the TiO_2_ film (Fig. 14).^42^ The obtained composite film was used for photocatalytic reduction of CO_2_, which showed extremely high reduction efficiency and CH_4_ selectivity. The work of Lin *et al.* shows the superiority of CdS loaded with Pt UMNPs in the photocatalytic hydrogen evolution reaction.^35^ They reduced Pt^4+^ into Pt UMNPs (1.75 nm) through ultrasonic radiation and deposited them on CdS nanorods. When the Pt loading was 0.5 wt%, the highest H_2_ release efficiency (24.15 mmol h^−1^ g^−1^) was achieved. The use of Pt UMNPs as co-catalysts on the surface of CdS can effectively promote the separation of photo-generated charges on CdS, offer a low activation potential, and provide active centers for H_2_ generation to enhance photocatalytic H_2_ evolution.

## 7. Synthesis of UMNPs supported by silica

Silica is one of the most abundant components in earth and possesses excellent chemical and thermal stability. It can be considered as an ideal candidate for catalyst supports because of its stability. Silica has a porous structure, especially for ordered mesoporous silica, which can disperse and isolate UMNPs to keep UMNPs stable during catalysis. There are also abundant silanol groups on the silica surface, which makes silica easy to be modified for anchoring UMNPs.^105^ Thus, silica has been widely used as supports for heterogeneous catalysts, especially in oxidation or reduction reactions driven by thermal energy such as semihydrogenation, CO oxidation, CH_4_ oxidation and so on.^106–108^

**Fig. 15:**
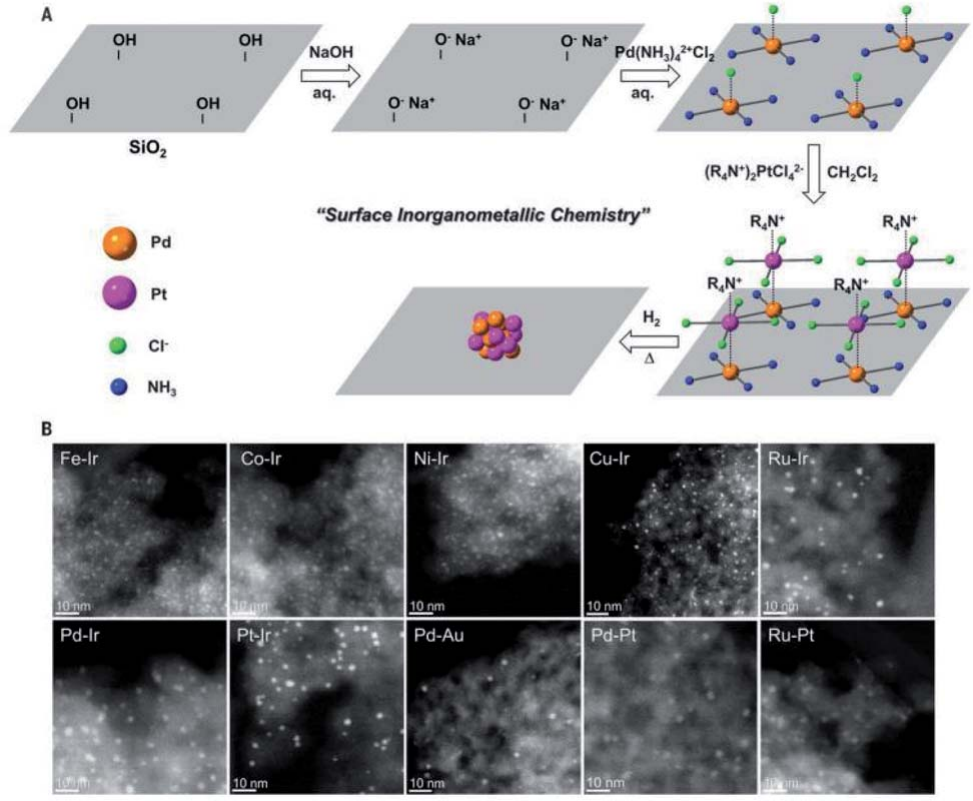
Schematic illustration and STEM of the supported bimetallic NPs (adapted with permission from ref. 106 @ copyright © 2018 American Association for the Advancement of Science).

Ding *et al.* synthesized a series of bimetallic UMNPs (1–3 nm) by decomposing and reducing metal salts adsorbed on silica (Fig. 15).^106^ These bimetallic UMNPs exhibit well-defined stoichiometry and intimacy between constituent metals and show excellent catalytic performance in semi-hydrogenation of alkynes. Fedorov *et al.* reported a highly efficient and selective catalyst for the semi-hydrogenation of alkynes, which is obtained by loading Cu UMNPs (2.0 ± 0.6 nm) on passivated silica.^109^ The competing passivation of silanol sites by Me_3_Si groups and low Cu precursor loading limit the density and size of Cu UMNPs on passivated silica. The size of Cu UMNPs and the IMes (1,3-bis(2,4,6-trimethylphenyl)imidazole-2-ylidene) anchored on the Cu surface play important roles in improving the selectivity for semihydrogenation.

Similar to mesoporous carbon, mesoporous silica can provide a suitable confined environment for UMNPs. Yang *et al.* reported a novel catalyst obtained by supporting Pd UMNPs (1.1 nm) on functional mesoporous silica nanoparticles.^110^ Not only the walls of silica can prevent the aggregation of UMNPs, but also the unevenly distributed functional groups in porous silica can maintain the UMNPs in the same channel dispersed. Besides, the isolated silanol groups may interact with phenol through hydrogen bonding and facilitate the phenol hydrogenation process. Kao *et al.* synthesized Ni UMNPs (2.7 nm) by using 2D hexagonal channel mesoporous silica (SBA-15C) and 3D cage-type mesoporous silica (SBA-16C) as supports.^111^ Such a composite catalyst exhibits high catalytic activity and stability in the hydrogenation of nitroarenes to aminoarenes.

**Fig. 16:**
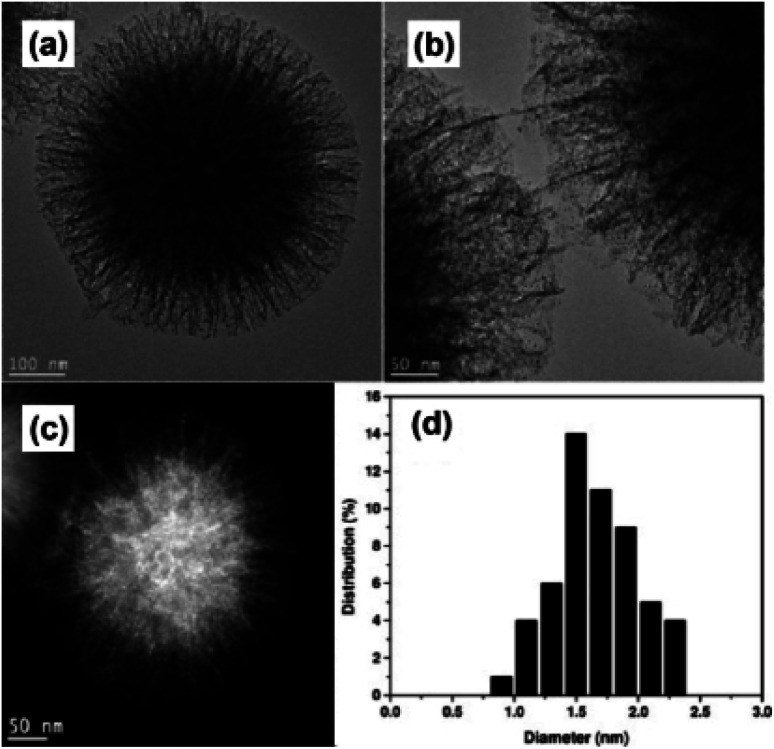
(a and b) HR-TEM images, (c) HAADF-STEM images and (d) size histogram of KCC-1-NH_2_ supported Au UMNPs (adapted with permission from ref. 107 @ copyright 2016 Wiley-VCH Verlag GmbH & Co. KGaA, Weinheim).

Even though mesoporous silica works as an efficient and stable supporting material for UMNPs, most UMNPs supported by mesoporous silica are trapped by pores near the surface rather than deeper pores inside, which may reduce the available surface area and may block the pores. As a unique type of silica, fibrous silica nanosphere (KCC-1) can provide a much higher surface area for UMNPs due to the fibrous morphology rather than pore structures.^112,113^ KCC-1 will not be affected by the blocking of pores and can provide faster mass transfer. Therefore, compared with mesoporous silica, KCC-1 is superior in various applications, including catalysis. Basset *et al.* used 3-aminopropyltriethoxysilane modified KCC-1 (KCC-1-NH_2_) as a support and introduced Au ions (Fig. 16).^107^ Then they obtained Au nanoparticles of different sizes through different reduction methods. Among them, Au UMNPs (1–2 nm) were obtained using NaBH_4_ as a reducing agent. It shows excellent catalytic activity for CO oxidation. The fibrous structure prevents the migration and aggregation of Au UMNPs, and also it provides a huge contact area with CO.

## 8. Conclusion and perspectives

In summary, controlling the synthesis of UMNPs through the use of solid supports can control the size of the UMNPs and help the UMNPs remain well-dispersed. During the catalytic process, the supports and the UMNPs can synergistically determine the catalytic activity. Therefore, synthesizing UMNPs *in situ* in the presence of a support and controlling the nucleation and growth of UMNPs is a promising method for the controlled synthesis and application of UMNPs in catalysis. The structure and properties of a support play an important role in the growth and application of UMNPs. Many organic porous materials, such as COFs, MOFs, OMCs, and CMPs, can be rationally designed at the atomic level, and their pore size and shape can be precisely controlled, so as to control the size and morphology of UMNPs. The ordered porous support structure is conducive to the narrow size distribution of UMNPs. Carbon materials such as porous carbon, hollow carbon, and graphene have excellent chemical and thermal stability. At the same time, because carbon materials are cheap and easy to obtain, they are economical support materials. Carbon materials doped with heteroatoms or modified with functional groups can provide a large number of nucleation and anchoring sites for UMNPs. The introduction of UMNPs into the vacancies or defects of metal oxides/sulfides can firmly bind UMNPs to the supports through strong coupling. In addition, UMNPs can be used as co-catalysts to enhance the photocatalytic activity of metal oxides/sulfides. The controlled growth of UMNPs mediated by solid supports has been summarized in Table 1, including different types of support materials, UMNPs with different types and sizes, and variable applications with catalytic activities.

**Table 1:** Controlled growth of UMNPs supports, size and application in catalysis

Type of supports	Support materials	Type of UMNPs	Design principles	Size	Types of catalysis	Catalytic activity	Ref.
Carbon materials	N-Doped porous carbon	Ni–Fe	Electronic effects	∼2 nm	Electrocatalysis (OER)	*η*_10_ = 297 mV	45
		Ni–Mo	Electronic effects	∼2 nm	Electrocatalysis (HER)	*η*_10_ = 130 mV	
	Hierarchically porous carbon	Pd	Electronic effects/spatial confinement effects	1.1 ± 0.2 nm	Dehydrogenation of formic acid	TOF achieved as 14 400 h^−1^ at 60 °C	114
	MOF-derived mesoporous carbon	H_2_O−WO_*x*_	Spatial confinement effects	∼2.24 nm	Epoxidation of *cis*-cyclooctene	TOF = 949 h^−1^	48
	MOF-derived mesoporous carbon	Pt	Spatial confinement effects	2–3 nm	Electrocatalysis (MOR)	1195 mA mg_Pt_^−1^	47
					4-Nitrophenol reduction	TOF = 0.200 s^−1^ g^−1^ L	
	MOF-derived porous carbon	Cu/Ru	Electronic effects/spatial confinement effects	3.30 ± 0.66 nm	Ammonia borane hydrolysis	TOF achieved as 97 mol_H_2__ mol_cat_^−1^ min^−1^	115
	3D N-doped porous carbon networks	Ru	Electronic effects/spatial confinement effects	∼2.47 nm	Electrocatalysis (HER)	*η*_10_ = 37 mV (0.5 M H_2_SO_4_), *etc.*	21
	N-Doped carbon nanosheets	Mo_2_C	Electronic effects	∼2 nm	Electrocatalysis (ORR)	*E*_1/2_ = 0.83 V	116
	3-D mesoporous graphene nanosheet	PtAg	Electronic effects/spatial confinement effects	∼2.5 nm	Electrocatalysis (ORR)	*E*_1/2_ = 0.92 V	117
	3-D N-doped graphene networks	Pt–Ni	Electronic effects	∼2.24 nm	Dehydrogenation of hydrous hydrazine	TOF = 943 h^−1^	118
	S-Doped graphitic carbon nitride	Ag	Electronic effects	<1 nm	Photocatalytic degradation of rhodamine B	Degradation rate is 96.5% after 50 min	119
	Nitrogen doped carbon nanosheets	Ru	Electronic effects/spatial confinement effects	∼1.41 nm	Ammonia borane hydrolysis	TOF achieved as 440 min^−1^	120
	MOF-derived carbon	Pt	Electronic effects/spatial confinement effects	2.1–4.1 nm	Electrocatalysis (HER)	*η*_10_ = 42.1 mV	28
	Hollow N-doped carbon tube	Ag	Electronic effects/spatial confinement effects	∼2.19 nm	CO_2_ conversion	Yield achieved as 99%	121
	PDIL–carbon nanotube	Pd_4_Au_1_–P	Electronic effects	∼2.3 nm	Electrocatalysis (EOR)	*I*_f_ = 2296.3 mA mg_Pd_^−1^	57
	N, P-Codoped hollow carbon nanospheres	Ru/Ni_2_P	Electronic effects	∼3 nm	Electrocatalysis (HER)	*η*_10_ = 89 mV (0.5 M H_2_SO_4_), *etc.*	122
	Mesocellular graphene network	Pd	Electronic effects	∼2.8 nm	Electrocatalysis (ORR)	*E*_1/2_ = 0.846 V	20
	Graphene oxide	Rh	Electronic effects	1.8 ± 0.4 nm	4-Nitrophenol reduction	*k* = 7.62 × 10^−3^ s^−1^, TOF = 18 min^−1^	37
	Reduced graphene oxide	Bi	Electronic effects	∼2 nm	Electrocatalysis (CO_2_RR)	FE (HCOOH) = 98%	43
	N-Doped carbon microtubes	Pd	Electronic effects/spatial confinement effects	∼2.3 nm	4-Nitrophenol reduction	TOF achieved as 29.5 min^−1^	58
					Suzuki coupling reaction	TOF achieved as 44.0 min^−1^	
	Reduced graphene oxide	Pt	Electronic effects	2–3 nm	Electrocatalysis (MOR)	46.43 A g_G-Pthybrids_^−1^	66
	Reduced graphene oxide	Cu	Electronic effects	2.0 ± 0.4 nm	4-Nitrophenol reduction	Conversion efficiency is 98.6%	67
	N-Doped carbon black embedded graphene	Pd	Electronic effects	∼3.2 nm	Electrocatalysis (EOR)	*I*_f_ = 2690.4 mA mg^−1^	68
Crystalline frameworks	MIL-101	CuPd	Electronic effects/spatial confinement effects	∼2.16 nm	Homocoupling reaction of phenylacetylene	Yield_(1,4-diphenylbuta-1,3-diyne)_ = 98%	24
	UiO-66	Pt–Co	Electronic effects/spatial confinement effects	∼2.0 nm	Hydrogenation of nitrobenzene	Conversion efficiency is more than 99%	123
	UiO-66	Pd	Electronic effects/spatial confinement effects	0.8–1.1 nm	Hydrogenation of benzoic acid	Yield_(cyclohexanecarboxylicacid)_ = 100%	76
	UiO-67	Pt	Electronic effects/spatial confinement effects	2.5 ± 0.7 nm	CO oxidation	TOF = 0.066 s^−1^	124
	UiO-66	Ni	Electronic effects/spatial confinement effects	∼2 nm	CO_2_ hydrogenation to methane	Yield_(methane)_ = 57.6%	13
	MOF199	Au	Electronic effects/spatial confinement effects	<2 nm	A3-Coupling reaction	Yield_(propargylamine)_ = 93%	125
	UiO-66-NH_2_, *etc.*	Pt	Electronic effects/spatial confinement effects	∼2 nm	CO oxidation reaction	Conversion efficiency is 100%	126
	2D nanosheet of mixed-ligand Ni(ii) MOF	Au	Electronic effects/spatial confinement effects	∼1 nm	4-Nitrophenol reduction	Total reacted to 4-aminophenol within 6 min, *k* = 0.404 min^−1^	72
	Zn-Based MOFs	Cu_2_O	Electronic effects/spatial confinement effects	1.61 ± 0.46 nm	Photocatalytic CO_2_ methanation	TOF = 50 × 10^−3^ s^−1^	127
	Multi-layered manner inside MOFs	Pt	Electronic effects/spatial confinement effects	∼3 nm	4-Nitrophenol reduction	*k* = 0.54 min^−1^, conversion efficiency is near 100%	128
	MOF-74	NiMg	Electronic effects/spatial confinement effects	0.7 ± 0.1 nm	Carbon dioxide methanation	Yield (CH_4_) = 55.8%	74
	Monolayered CoN_4_-based MOF	CoFeO_*x*_	Electronic effects/spatial confinement effects	∼3 nm	Electrocatalysis (OER)	*η*_10_ = 232 mV	77
	Nitrogen-rich COFs	Ru	Electronic effects/spatial confinement effects	1.4–2.6 nm	Methanolysis of ammonia borane	TOF achieved as 505 min^−1^	80
	Triazine basic COFs	Pt	Electronic effects/spatial confinement effects	∼2.10 nm	Electrocatalysis (ORR)	*E*_1/2_ = 0.89 V	129
	sp^3^ N-rich flexible COF	Co_*x*_Ni_*y*_(OH)_2_	Electronic effects/spatial confinement effects	∼2 nm	Electrocatalysis (OER)	*η*_10_ = 258 mV	130
	Phosphine-based COFs	Pd	Electronic effects/spatial confinement effects	∼1.62 nm	Suzuki–Miyaura coupling reaction	TOF achieved as 1648 h^−1^	33
	Phenol–pyridyl COF	Cu/Cu_2_O	Electronic effects/spatial confinement effects	2–3 nm	Glaser–Hay coupling	Yield achieved as 80%, TOF = 50 h^−1^	131
	TpBD–Me_2_ COF	RuO_2_	Electronic effects/spatial confinement effects	∼1.2 nm	Formic acid dehydrogenation reaction	Yield of H_2_ achieved as 97%	132
	TpTa–COF	Fe doped TiO_2_	Electronic effects/spatial confinement effects	2.3 ± 0.9 nm	Photocatalytic degradation of methylene blue	Degradation efficiency over 95%	19
	Amine-functionalized COFs	Pd	Electronic effects/spatial confinement effects	1.58 ± 0.2 nm	Benzyl alcohol oxidation	97.0% selectivity to benzaldehyde	85
	Sulfur-containing covalent organic framework	Au	Electronic effects/spatial confinement effects	4.2 ± 1.2 nm	4-Nitrophenol reduction	Total reacted to 4-aminophenol within 7 min	36
	COFs with thiol chains	Au	Electronic effects/spatial confinement effects	1.8 ± 0.2 nm	Photocatalytic degradation of RhB	Degradation achieved as 97.3%	84
					Photocatalytic degradation of bisphenol A	Degradation more than 90%	
	Thioether-containing COFs	Pt	Electronic effects/spatial confinement effects	1.70 ± 0.2 nm	4-Nitrophenol reduction	Total reacted to 4-aminophenol within 8 min	25
		Pd	Electronic effects/spatial confinement effects	1.78 ± 0.2 nm	Suzuki–Miyaura coupling reaction	Yield is more than 99.0%	
Amorphous organic polymers	Redox-active CMPs	Au	Electronic effects/spatial confinement effects	∼2 nm	Reduction of nitro aryls	Yield is more than 99.0%	89
		Co	Electronic effects/spatial confinement effects	∼10 nm	Electrocatalysis (ORR)	*E*_onset_ = 0.91 V (*vs.* RHE)	
	Electron-rich 3D CMP	Pd	Electronic effects/spatial confinement effects	∼2.4 nm	Suzuki coupling reaction	Yield achieved as 96%	90
					Sonogashira cross-coupling reaction	Yield achieved as 96%	
					Stille cross-coupling reaction	Yield achieved as 97%	
	Covalent carbazole framework	Ag	Electronic effects/spatial confinement effects	∼2.5 nm	4-Nitrophenol reduction	Normalized rate constant achieved as 21.49 mmol^−1^ s^−1^	133
	Aminal-based HCPs	Pd	Electronic effects/spatial confinement effects	∼2.89 nm	Reduction of nitroarenes	Yield achieved as 99%	93
	Mesoporous HCPs	Bi	Electronic effects/spatial confinement effects	1–3 nm	4-Nitrophenol reduction	Reaction rate constant *k* = 2.2 min^−1^	134
	Hypercrosslinked polystyrene	Pt	Spatial confinement effects	2.3 ± 0.5 nm	Catalytic in wet air oxidation of phenol	Conversion efficiency is 97%, selectivity to CO_2_ and H_2_O is 94.2%	135
	HCP–PPh_3_	Rh	Electronic effects/spatial confinement effects	∼2.1 nm	Ammonia borane hydrolysis	TOF = 481 mol_H_2__ (mol_Rh_ min)^−1^	136
	HCPs	Au	Spatial confinement effects	1.7–5.1 nm	Reduction of 4-nitrophenol	Conversion efficiency is near 100%	22
	PyPPh_2_@POP	Pd	Electronic effects/spatial confinement effects	1.81–3.40 nm	Dehydrogenation of 3-methyl-2-cyclohexen-1-one to 3-methyl phenol	Conversion efficiency is 88.2%	137
	Phosphorus-doped POPs	Pd	Electronic effects/spatial confinement effects	∼2.7 nm	Hydrogenation of α, β-unsaturated compound	Yield achieved as 99%	138
					Reductive cyclization of 2-nitrophenylacetonitrile to indoles	Yield achieved as 99%	
	Triazinyl-containing POP	Pt	Electronic effects/spatial confinement effects	∼2.96 nm	Ammonia borane hydrolysis	TOF = 133.17 mol_H_2__ mol_Pt_^−1^ min^−1^	139
					Hydrogenation of halogenated nitrobenzenes	Conversion efficiency is 100% with a selectivity of 98%	
	Gallic acid-derived POPs	Ag	Electronic effects/spatial confinement effects	∼1 nm	Carboxylative cyclization of CO_2_ and propargyl alcohols	Yield achieved as 99%	140
	Triazine functionalized POP	Pd	Electronic effects/spatial confinement effects	∼3 nm	Alkene hydrogenation	Conversion efficiency is 99%	141
	Prefunctionalized POPs	Pd	Electronic effects/spatial confinement effects	1.6 ± 0.35 and 3.5 ± 0.35 nm	Dehalogenation reaction of chlorobenzene	Yield is near 100%	142
	Porous magnetic core–shell POP nanospheres	Pd	Electronic effects/spatial confinement effects	1.5–2.1 nm	Hydrogenation of nitrobenzene	Yield is 100%, TOF = 106.4 h^−1^	11
					Hydrogenation of alkenes and alkynes	Yield is 100%, TOF = 212.8 h^−1^	
	POPs	Pd	Electronic effects/spatial confinement effects	0.9–4 nm	Suzuki–Miyaura coupling reaction	Yield achieved as 92%	143
					Sonogashira coupling reaction	Yield achieved as 99%	
	1,2,3-Triazolyl-containing POPs	Pd	Electronic effects/spatial confinement effects	1.39 ± 0.31 nm	Hydrogenation of 1-hexene	Yield is 100%	144
	Boron organic polymers	Pt	Electronic effects/spatial confinement effects	∼2.3 nm	Ammonia borane hydrolysis	TOF = 1082.5 mol_H_2__ mol_Pt_^−1^ min^−1^	145
		Pd	Electronic effects/spatial confinement effects	∼3.6 nm	Ammonia borane hydrolysis	TOF = 890.0 mol_H_2__ mol_Pd_^−1^ min^−1^	
	Imidazolium-based organic polymers	Pd–Au	Electronic effects/spatial confinement effects	1.50 ± 0.20 nm	Ammonia borane hydrolysis	25.0 mol_H_2__ mol_cat_^−1^ min^−1^	146
	TB–POP	Pd	Electronic effects/spatial confinement effects	1.5 ± 0.6 nm	Dehydrogenation of formic acid	TOF achieved as 1344 h^−1^, selectivity is 100%	31
	Triazinyl-pentaerythritol POPs	Pd	Electronic effects/spatial confinement effects	1.4–2.8 nm	4-Nitrophenol reduction	Yield is more than 99%	32
Metal oxide/sulfide	CeO_2_ nanorods	Pt	Electronic effects	1–2 nm	4-Nitrophenol reduction	Similar as surface-embedded sample	98
	CeO_2_ nanorods	Pt	Electronic effects	1.3–2.5 nm	Oxidation of toluene	TOF_Pt_ = (7.95 ± 0.43) × 10^−3^ s^−1^	147
	CeO_2_ nanorods	Pd	Electronic effects	∼2 nm	CO oxidation	Generation rate is 1 × 10^21^_molecules CO_ g_Pd_^−1^ s^−1^	148
	CeO_2_ nanocrystals	Au	Electronic effects	∼3 nm	CO oxidation	TOF = 0.69 s^−1^	149
	Mesoporous CeO_2_	Au	Electronic effects/spatial confinement effects	∼3 nm	CO oxidation	The initial conversion is of *ca.* 40%	95
	Ce–MOFs derived CeO_2_ nanoparticles	Pt	Electronic effects	<2 nm	CO oxidation	Conversion efficiency above 90% (170 °C), and 100% (650 °C)	34
	Porous CeO_2_ nanofibers	Pt	Electronic effects	∼1.7 nm	Water gas shift reaction	CO conversion reached 95% under 450 °C	150
	3D ordered macroporous TiO_2_	Pd	Electronic effects/spatial confinement effects	∼1.1 nm	Soot oxidation	Yield of CO_2_ = 97.6%	151
	N-Doped TiO_2_	Pd	Electronic effects	∼2.2 nm	H_2_O_2_ synthesis	H_2_O_2_ productivity = 4.1 mol_H_2_O_2__ g_Pd_^−1^ h^−1^	152
	TiO_2_ nanoparticles	Cu	Electronic effects	2–4 nm	Photocatalytic hydrogen generation	Generation rate of H_2_ = 9.5 mmol g^−1^ h^−1^	153
	TiO_2_	Pd	Electronic effects	1–2 nm	Photocatalytic hydrogen peroxide production	Selectivity >80%	154
	TiO_2_ single crystals	Pt	Electronic effects	0.5–2 nm	Photocatalytic CO_2_ photoreduction	CH_4_ yield is 1361 μmol g_cat_^−1^ h^−1^	42
	Co_3_O_4_ microflowers	Pt	Electronic effects	∼2.3 nm	Electrocatalysis (HER)	*η*_10_ = 34 mV	155
	CdS nanorods	Pt	Electronic effects	∼1.75 nm	Photocatalytic hydrogen evolution	H_2_ evolution rate of 24.15 mmol h^−1^ g^−1^	35
	Hollow CdS structure	Pd/PdS	Electronic effects	∼1.5 nm	Photocatalytic hydrogen evolution	H_2_ evolution rate of up to 144.8 mmol h^−1^ g^−1^	156
	MoS_2_ nanosheets	Pt	Electronic effects	2–5 nm	Electrocatalysis (HER)	*η*_10_ = 31 mV	157
	CoS2 nanosheet	Pt	Electronic effects	∼1.7 nm	Electrocatalysis (HER)	*η*_10_ = 24 mV	158
					Electrocatalysis (OER)	*η*_10_ = 300 mV	
	Mesoporous TiO_2_	RuO_2_	Electronic effects/spatial confinement effects	∼2 nm	CO_2_ methanation	2.05 μmol_CH_4__ g_cat_^−1^·s^−1^	159
	Urchin-like mesoporous TiO_2_ hollow spheres	Pt	Electronic effects/spatial confinement effects	∼3.2 nm	Electrocatalysis (ORR)	*E*_1/2_ = 0.867 V	102
	Mesoporous Co_3_O_4_	Pt	Electronic effects/spatial confinement effects	1.1–2.1 nm	Oxidation of methylene blue	*K*_35°C_ = (9.23 ± 0.15) × 10^−4^	103
	3D ordered mesoporous Co_3_O_4_	Au–Pd	Electronic effects/spatial confinement effects	2.7–4.5 nm	Methane combustion	*T*_90_ = 324 °C	104
Silica	2D silica nanosheets	Ag	Electronic effects	∼2.77 nm	Reduction of 4-nitrophenol	TOF = 3.52 min^−1^	160
	Mesoporous silica nanoparticle	Pd	Electronic effects/spatial confinement effects	0.9 ± 0.2 nm	Reduction of 4-nitrophenol	*k* = 0.31 min^−1^	161
	Cage-type mesoporous silica	Ni	Electronic effects/spatial confinement effects	∼2.7 nm	CO_2_ hydrogenation	TOF_CO_2__ >110 s^−1^	162
	Silica	Ru	Electronic effects/spatial confinement effects	∼1.56 nm	CO_2_ methanation	CO_2_ conversion achieve as 70%	163
	Silica	Pt–Pd	Electronic effects/spatial confinement effects	1–3 nm	Semihydrogenation of alkynes	C_2_H_2_ conversion achieve as 100% near 100 °C	106
	Passivation silica	Cu	Electronic effects/spatial confinement effects	2.0 ± 0.6 nm	Semihydrogenation of alkynes	Selectivity to (*Z*)-olefins achieve as 97%	109
	Bimodal mesopore silica	Pd	Electronic effects/spatial confinement effects	∼3 nm	Oxidation of toluene	*T*_90_ = 228 °C	108
	–COOH functionalized SBA-16	Ag-Doped Ni	Electronic effects/spatial confinement effects	∼3 nm	Reduction of 4-nitrophenol	Activity parameter achieve as 3340.4 s^−1^ g_AgNi_^−1^	164
	Mesoporous silica	RhO_*x*_	Electronic effects/spatial confinement effects	1–2.5 nm	N_2_O decomposition	*T*_90_ = 407 °C	165
	SBA-15 monoliths	Ni	Electronic effects/spatial confinement effects	1–3 nm	Methane dry reforming	TOF = 1.4 s^−1^	166
	Mesoporous silica	Au–Cu	Electronic effects/spatial confinement effects	∼1.5 nm	Glycerol oxidation	Dihydroxyacetone selectivity = 90%	167
	3D dendritic mesoporous silica nanospheres	Pd	Electronic effects/spatial confinement effects	∼1.5 nm	Suzuki–Miyaura cross-coupling reactions	Conversion >99%	168
	Mesoporous MMT-1 silica	Pd	Electronic effects/spatial confinement effects	∼1.1 nm	Hydrogenation of phenol to cyclohexanone	Conversion = 99%, selectivity = 98%	110
	Ordered mesoporous silicas	Ni	Electronic effects/spatial confinement effects	∼2.7 nm	Hydrogenation of nitroarenes	Apparent reaction rate = 5.68 × 10^−3^	111
	PDETA-functionalized KCC-1	Pd	Electronic effects/spatial confinement effects	∼2.8 nm	Dehydrogenation of formic acid	TOF = 332 h^−1^, selectivity_H_2__ = 100%	169
	Aminopropyl groups functionalized KCC-1	Pd	Electronic effects/spatial confinement effects	1–5 nm	Suzuki coupling reaction	Yield achieved as 97%	170
	Aminopropyl groups functionalized KCC-1	Au	Electronic effects/spatial confinement effects	1–2 nm	CO oxidation	*T*_50_ = 275 °C	107

This review summarizes the control of the growth of UMNPs with solid supports and their applications in the field of catalysis. Through the analysis of the reported examples, we believe that the following conditions play crucial roles in controlling the growth of UMNPs, while still facing some problems and challenges:

(1) The design of supports: porous supports can provide confinement for the growth of UMNPs and prevent the accumulation of UMNPs through site isolation. At the same time, the high specific surface area provides a large number of available active sites and anchoring sites. However, in many cases, UMNPs grow outside of the pores of the porous support, which causes the pores to be blocked. Many active sites in the pores cannot participate in the catalytic process, which affects the mass transfer of the catalytic process. Through a proper design, metal precursors can be preferentially loaded inside the pores, which can effectively prevent the growth of UMNPs on the surface of supports. The hierarchical design of micropores, mesopores, and macropores can incorporate the micropores and smaller mesopores, which may help in efficient transfer mass. It is also necessary to consider the interactions between supports and UMNPs to facilitate catalytic reactions while avoiding catalyst poisoning.

(2) Synergistic catalysis provided by UMNPs and supports: some supports can adsorb reaction substrates, which increases the local concentration of reaction substrates near UMNPs and facilitates the catalytic reactions. Bai *et al.* prepared covalent triazine framework (CTF) nanosheets coated with Ag particles to catalyze CO_2_ conversion.^171^ CTFs have no obvious catalytic activity for CO_2_ conversion, but they have high CO_2_ capture ability. The coated Ag particles can catalyze the coupling of CO_2_ and phenylacetylene to produce 3-phenylpropiolic acid. The hybrid materials of Ag particles and CTFs can act synergistically to catalyze the reaction. Through the rational design of UMNPs and supports, the catalytic activity of the catalyst can be improved effectively.

(3) Shape and diameter of UMNPs: precise control of the shape and diameter of UMNPs is still challenging, which is critical to improving their performance in heterogeneous catalysis. The spatial confinement effect is more inclined to macroscopic design, which can limit the growth of nanoparticles and control the size and shape of nanoparticles to a certain extent. It is still hard to control UMNPs with a size less than 3 nm by using most of the inorganic supports, while electronic effects have a remarkable influence over the size of UMNPs. The size of UMNPs can be fine-tuned by adjusting the concentration of metal ions and the ratio of heteroatoms, functional groups or defects. The development of new supports that can combine the electronic effects and characteristic spatial shapes may be the future of realizing the precise control of UMNPs, especially for the shape control.

(4) Stability: high stability of the UMNPs and carriers, even under harsh catalytic conditions, is highly desired. Improving the stability of the carrier and UMNPs can increase the number of cycles of a catalyst, which is conducive to reducing the cost of the catalyst and enabling UMNPs for practical applications. Long-term usage or harsh conditions (such as high temperature or corrosive medium) may ruin the structure of solid supports and cause the catalyst to be deactivated. It calls for the development of more stable or even self-repairable supports.

(5) Nucleation and growth mechanism: there have been some studies on the nucleation and growth mechanism of UMNPs on the carrier interface, but the mechanism of nucleation and growth is still unclear and in-depth exploration is needed, which can provide design principles of solid supports and efficient synthetic routes to MNPs.

Although great progress has been achieved, controlling the nucleation and growth of UMNPs through the use of solid supports still faces many challenges. With continuous in-depth research, efficient preparation of UMNPs with well-controlled uniform size and high stability is anticipated, which will enable wide-spread practical applications of UMNPs in the field of catalysis and beyond.

## Conflicts of interest

There are no conflicts to declare.

## Supplementary Material
